# Conscientious objection and barriers to abortion within a specific regional context - an expert interview study

**DOI:** 10.1186/s12910-024-01007-1

**Published:** 2024-02-06

**Authors:** Robin Krawutschke, Tania Pastrana, Dagmar Schmitz

**Affiliations:** 1 Institute for History, Theory and Ethics in Medicine, RWTH Aachen University, Wendlingweg 2, Aachen, D-52074 Germany; 2 Department of Palliative Medicine, Medical Faculty, RWTH Aachen University, Pauwelsstraße 30, Aachen, D-52074 Germany

**Keywords:** Abortion, Conscientious objection, Reproductive autonomy

## Abstract

**Background:**

While most countries that allow abortion on women’s request also grant physicians a right to conscientious objection (CO), this has proven to constitute a potential barrier to abortion access. Conscientious objection is regarded as an understudied phenomenon the effects of which have not yet been examined in Germany. Based on expert interviews, this study aims to exemplarily reconstruct the processes of abortion in a mid-sized city in Germany, and to identify potential effects of conscientious objection.

**Methods:**

Five semi-structured interviews with experts from all instances involved have been conducted in April 2020. The experts gave an insight into the medical care structures with regard to abortion procedures, the application and manifestations of conscientious objection in medical practice, and its impact on the care of pregnant women. A content analysis of the transcribed interviews was performed.

**Results:**

Both the procedural processes and the effects of conscientious objection are reported to differ significantly between early abortions performed before the 12th week of pregnancy and late abortions performed at the second and third trimester. Conscientious objection shows structural consequences as it is experienced to further reduce the number of possible providers, especially for early abortions. On the individual level of the doctor-patient relationship, the experts confirmed the neutrality and patient-orientation of the vast majority of doctors. Still, it is especially late abortions that seem to be vulnerable to barriers imposed by conscientious objection in individual medical encounters.

**Conclusion:**

Our findings indicate that conscientious objection possibly imposes barriers to both early and late abortion provision and especially in the last procedural steps, which from an ethical point of view is especially problematic. To oblige hospitals to partake in abortion provision in Germany has the potential to prevent negative impacts of conscientious objection on women’s rights on an individual as well as on a structural level.

**Supplementary Information:**

The online version contains supplementary material available at 10.1186/s12910-024-01007-1.

## Background

Since abortions have been decriminalized and new laws on abortions have been enacted in many countries worldwide over the last decades, lawmakers faced a challenge to balance women’s rights to healthcare and reproductive self-determination [1] with health workers’ right to freedom of thought, conscience and religion [2]. Reproductive self-determination, also termed as “reproductive autonomy” is variously understood, but generally means not only that women or couples should be able to make their reproductive decisions free from coercion, discrimination and violence. It encompasses in a wider sense a “right to control [one’s] own role in procreation unless the state has a compelling reason for denying them that control” [3]. Most countries which allow abortions on women’s request or under common legal grounds (e.g. if the pregnancy is a result of rape) have included an addendum to their new jurisdictions that grants physicians and other health professionals the option to refrain from performing them on the grounds of moral bias [4]. This so-called conscientious objection (CO) can be defined based on the UN’s International Covenant on Civil and Political Rights [2] as the refusal to “act according to a legal mandate or obligation, or an administrative order” [5] “that an individual considers incompatible with his/her religious, moral, philosophical or ethic beliefs” [6]. CO is thus supposed to protect the moral integrity and personal autonomy of health care providers [7]. However, as studies have shown, it seems to harbour the potential to constitute a serious and multi-layered barrier for women seeking abortion [8, 9] which is why an ethical debate has risen among scholars whether CO could indeed be a *compelling reason* for restricting reproductive autonomy.

Several studies from Latin America [5, 10–13], Australia [14] and Europe [15–17] have indicated that it might have harmful consequences for women’s health if CO practice is not regulated or if objectors misuse it or do not act according to applicable guidelines [4, 9, 18, 19]. Critics like Fiala et al. advocate a position of non-tolerance towards CO and argue that claims for CO are “non-verifiable” and “subjective” [20] and call CO a “dishonourable disobedience, because it violates women’s fundamental right to lawful healthcare” [21]. Savulescu states that “a doctor’s conscience has little place in the delivery of modern medical care” because CO corrupts the delivery of treatments that are legally permitted and beneficial [22]. CO to abortion is, thus, criticized with regard to beneficence-based as well as autonomy-based obligations of health professionals.

On the other hand, advocates like Blackshaw counter that abortions are neither demonstrably beneficial for women’s health, nor are they inevitably clinically indicated and should therefore qualify for CO [23]. Wicclair emphasises that CO’s individual value and importance for health professionals’ moral integrity should not be undermined. He argues that, despite concerns about the potential impact of CO on patients, other professionals and health care institutions, there must be irrefutable reasons to legitimise a non-accommodation or ban on CO in healthcare. The proponents of such a ban have not yet been able to present these, which is why he favours a position of reasonable accommodation [24]. This is an extension of the so-called ‘conventional compromise’ developed by Dan Brock, which is intended to clarify the conditions under which a physician’s CO is compatible with his or her professional duties and balance those duties to the public with “protecting the individual professional’s moral integrity” [25] (p. 196). Both approaches essentially include that objectors inform their patients of their conflict of conscience, but nevertheless provide patients with information, counsel them on the treatment options available and refer them to a willing provider in a timely manner. Wicclair adds that accommodation of CO “should not impose excessive burdens on other clinicians, administrators, or organizations” [26] (p. 94). As correct and important as these considerations are for finding a compromise, they are formulated in a vague manner in order to take into account the various possible conflict situations and thus offer few tangible solutions for regulating CO in real practice.

CO is regarded as an understudied phenomenon [8]. As most countries do not require physicians to register their decision if objecting against performing an abortion, there is only limited data on the prevalence of CO. Still, some existing studies show that up to around 90% (Mexico) [5] or even more than 90% (Italy - with regional differences) [27] of the physicians declare themselves conscientious objectors and refuse to perform abortions. Despite it being a global phenomenon, it is expected to have very variable regional effects and should therefore also be examined in specific local contexts [6, 28]. So far, few studies have perused how CO actually impacts on abortion processes in secular states in Central Europe [16, 17]. A multiple-case study comparing the approaches to regulating CO in England, Italy, Norway and Portugal analysed each country’s attempt to balance the contending rights of women and health care professionals and found that by ‘imposing constraints on objectors and by assuring ready access (..) it is possible to permit CO to abortion and still ensure (…) access to care’ [15]. For Germany, the available data on CO is old and inconclusive. A non-representative study from 1986, for example, found out that 40% of the 406 interviewed gynaecologists offered abortion services while more than 40% opposed abortion as an intentional killing of human life. Professional or personal moral beliefs were seen as the second most important causes for not offering abortion services [29]. A correlation between self-appointed higher religiosity and a higher rate to disapprove or object to abortions could also be identified in Denmark [30] and the United States [31]. According to the Federal Statistical Office, the number of abortion providers in Germany decreased by 46,7% from 2003 [32] to 2021 [33]. Due to missing data, we have no knowledge regarding the causes for this decrease. Against this background, however, it seems to be all the more important to clarify possible manifestations of CO to abortions.

This paper aims to reconstruct how abortions are organised in a mid-sized German city (about 250,000 inhabitants) and the ways in which CO might affect abortion provision. Thereby we distinguish those abortions that are being performed during the first trimester of pregnancy (to which we refer from here on as ‘early abortions’) from abortion provision based on medical indications after the first trimester (to which we refer as ‘late abortions’). Since abortions are ultimately performed by doctors, and in this case gynaecologists, we will focus primarily on doctors’ CO. This should not obscure the fact that CO also occurs in other professional groups, such as nurses or pharmacists, and might also have a significant influence on medical practice and patient care.

## Methods

A qualitative study design using expert interviews was adopted to gather first-hand information about local structures and to understand perceptions and activities of medical professionals in their real-life settings. An individual was considered an expert provided that he or she is directly involved in either performing abortions or examining, referring, or counselling women in order to arrange an abortion.

We used a purposive sampling strategy combining network-based and snowball approaches just as word-of-mouth recommendations. Thereby, all professional groups which were ex-ante attributed with a pivotal role in abortion provision were questioned. Accordingly, one expert from a counselling centre, two registered gynaecologists (one of which provides abortion services), one midwife and one hospital doctor were interviewed. The candidates were invited by mail to participate in our qualitative interview-based study and informed about the aim of the study. All contacted experts accepted the invitation.

A semistructured interview guide was elaborated based on the literature research. The guide included questions about medical care structures with regard to abortion procedures, the application and manifestations of CO in medical practice, and its impact on the care of pregnant women (see supplementary file). Participating experts were also asked to raise further issues which they deemed relevant in the context of the study.

The five interviews themselves were conducted by RK in April 2020. One of the interviews was held by telephone (due to the pandemic situation at that time), while the other four took place in person at the participant’s respective workplace, at a convenient time as agreed with the interviewees. All interviews were conducted and analysed in German. Selected quotes were translated for this article. Interviews were audio-recorded and lasted between 45 and 60 minutes. The participants of the study gave verbal informed consent to be recorded and approved the use for further analysis. All of the recorded interviews were transcribed verbatim by RK and were not returned to the participants. Audio tapes were deleted after anonymized transcription.

A content analysis of the transcripts was performed using the software MAXQDA 20.3. In a first step, a-priori categories were based on the interview guide. Furthermore, themes within each respective category were identified inductively. Thereby, a coding system was developed that was consequently used for analysis. Eventually, word processing software was applied to compile analysis findings.

We ensured scientific rigor through regular cross-checking between researchers at every stage of the research.

## Results

We organize our findings along two dimensions which were derived from the content analysis: 1) medical care structure with regard to abortions and 2) possible manifestations of CO.

All interview participants were involved in either early or late abortion services, none in both fields. All were aware of the prerequisites under which the “other field” operates, but how exactly and by whom those abortion services are being offered was beyond the knowledge of most interviewees.

### Medical care structure and processes of early abortions

**Fig. 1:**
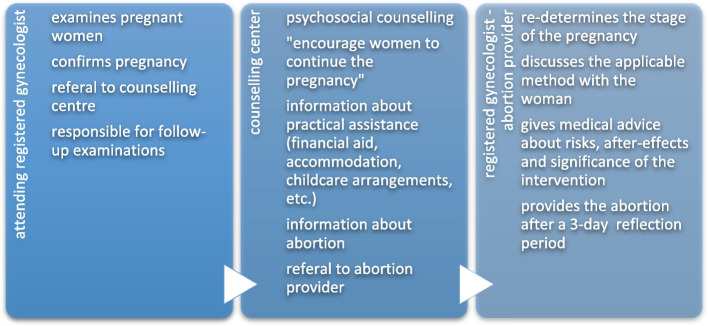
Relevant agents and their responsibilities in early abortions

A typical pathway prior to an early abortion, as shown in Fig. 1, involves three professional agents:

Abortions in this town are performed by four gynaecologists: two offer both surgical and medical abortions and another two only perform the latter. Further three gynaecologists located in the suburbs also offer medical abortions. Thus, two or seven doctors - depending on the respective method - bear the responsibility for abortion services in the region. The number of abortion providers is not monitored by any political authority. Instead, gynaecologists themselves seem to ensure that a sufficient number of gynaecologists willing to continue abortion services is being maintained.

*‘I know that the one colleague who has now taken over the practice was specially chosen by her predecessor on the premise that she would do abortions. (…) That was the condition, so to speak, to hand over the practice, because she said: “I want to be sure that it is guaranteed that someone will do it when I am no longer practicing”. In this respect, it is not the city that is interested, but the retiring doctor ensures that someone new does it.’ (female registered gynaecologist, not performing abortions, 2nd interview).*

In the region under study, early abortions are exclusively performed by registered gynaecologists in their offices. Hospitals are only involved if post-procedural complications occur.

### Manifestations of conscientious objection in early abortions

The interviews revealed that CO is a multifaceted phenomenon that potentially has different impacts at different levels of health care provision: CO (1) has a direct influence on the individual professional practice and the physician-patient interaction. But it also might (2) impact on regional care structures like the general number of providers available for abortions.

For the individual aspect, most interviewees agreed that in the majority of cases of CO in early abortions, the doctor acted in accordance with the established guidelines [9, 18, 19]. Doctors mostly seem to refer their patients promptly and do not let their conscientious bias interfere in the process.

*‘My experience is that this really runs absolutely smoothly.‘(male registered doctor, performing abortions, 5th interview).*

Yet, there have been scattered reports about health care professionals involved in the structures described above who for example deliberately delayed abortion provision.

*‘I sometimes made the experience that women consult their attending gynaecologist very early in the pregnancy and if it is someone who opposes abortions, he or she first says: “I cannot see anything yet, you will have to come back in a fortnight.” Two weeks later they then say: “Yes, well, we will have to check that again.” And if the women do not come to a counselling centre of their own accord, the possibility of an abortion may already have passed. I sometimes have the impression that those doctors really actively try to delay the women in the hope that it will be too late at some point.’ (female counsellor, 1st interview).*

With regard to structural consequences, CO to early abortion provision according to the interviewees mainly affects the medical care structure by further reducing the number of abortion providers and assisting personnel.

Upon request the German health insurance company AOK reported that there are 39 gynaecological practices with 58 gynaecologists in the town, of which, following our findings, four gynaecologists offer abortions. We do not have any knowledge about the individual causes for not offering abortions, but (as cited above) some evidence from older national and international studies suggests that professional or personal moral beliefs do play an important role.

Despite the small number of practices offering abortion services, the experts mostly considered the medical care situation to be sufficient with regard to early abortion.

*‘We are in a very comfortable situation here. (…) Abortion provision here is well ensured.’ (female counsellor, 1st interview).*

Especially in suburban and rural areas a scarcity of resources can lead to a gap in healthcare provision. As a consequence, the four practices mentioned above receive patients not only from the examined city but from a larger area that extends up to 65 km. The underserved healthcare situation in rural areas entails that women need to put up with considerably more effort and invest more time in order to find a gynaecologist who is willing to perform the abortion. The additional time necessary might even lead to women having difficulties to follow through on the termination within the licit time limits.

Some participants also expressed their criticism that none of the local hospitals performs early abortions. It is especially Catholic, but also municipal hospitals who regularly refuse early abortion provision as a whole institution on grounds of conflict with religious beliefs or the organisational culture, regardless of whether some of their doctors would be willing to perform them.

*‘Formally, it is an individual thing, but if you send someone to hospital and they immediately refuse or say: “You don’t need to send anyone here because we don’t do that”, then it is de facto only the institution that objects and not the individual.’ (male registered gynaecologist, performing abortions, 5th interview).*

In conclusion, CO on an individual level mainly occurs in the first step of the pathway for early abortions and complicates women’s access to the following steps, whereas CO on a structural level plays out on the last step of the process by diminishing the number of providers (doctors and hospitals).

### Medical care structure and processes of late abortions

**Fig. 2:**
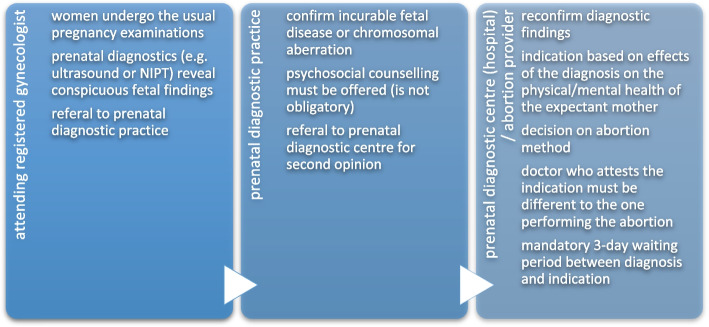
Relevant agents and their responsibilities in late abortions

The typical pathway prior to a late abortion is portrayed in Fig. 2. It differs significantly from that of early abortions, mainly because the pregnancies are initially wanted.

After the first trimester, ambulatory terminations are not performed in Germany. Therefore, registered gynaecologists are no longer in a position to perform these interventions and women in need must consult local hospitals in and around the town of which only one offers abortion services for second and third trimester pregnancies.

Depending on the gestational age and the potential viability of the foetus, a drug-induced induction with or without a foeticide may be considered to terminate the pregnancy. While the induction of an abortion by administration of prostaglandin can basically be carried out by all gynaecologists, only one physician is willing to perform foeticides in the respective hospital. Midwifes are also directly involved in late abortions and foeticides. They accompany and care for the women during the expulsion of the foetus or if complications occur. In contrast to gynaecologists, they must ensure their willingness to participate in late abortions before employment. Participation is thereby defined as either hands-on patient care or at least through documentation work and follow-ups.

### Manifestations of conscientious objection in late abortions

CO in late abortions is also expected to have an impact on both individual patient – professional interactions and regional care structures. Hospitals that provide abortions usually have adapted personnel structures and specifically appointed gynaecologists to take charge of these interventions. Accordingly, the women usually are not cared for by objecting staff, as stressed by the hospital officials:

*‘We are very careful not to create any feeling that leads women to doubt whether they have made the right choice. You know, that’s not our job anymore. (...) We only listen, we don’t discuss.’ (female midwife, performing abortions, 3rd interview).*

Nevertheless, here too, some interview participants referred to hospital staff who openly disapproved of their patient’s decision:

*‘Once, there was a hospital in the region where the chief physician agreed to perform abortions. But the staff at the registration desk, on the ward or in the operating theatre were so unfriendly that the women referred to it as a horrible and very unpleasant experience.’ (female counsellor, 1st interview).*

Although a medical indication is not based on the malformation of the foetus but on the danger to the health of the pregnant woman, gynaecologists sometimes refuse to perform late abortions if they do not consider the malformation to be significant enough. Thus, late abortions are at times also objected to on an individual basis and then affect the pregnant woman during the very last step of the process.

Whenever this objecting doctor is in an executive position, this individual CO also has far-reaching structural consequences, as it means that the hospital as a whole ceases to be a provider. In addition, some cases where reported, where smaller catholic clinics attested the medical indication and an abortion would also have been possible on the part of the doctors practicing there. Nevertheless, the abortion was rejected by the hospitals’ administration with reference to the catholic background and thus institutional CO has been claimed.

Problems arise when even larger clinics (or single leading professionals in these larger clinics) refuse to perform late abortions. Only a few years ago, none of the local hospitals in the surveyed region was willing to perform abortions. Women either decided to go abroad or they had to be sent to other hospitals about 40 to 100 km away. There, the workload became so great due to the enlarged catchment area that some of the women were re-referred again:

*‘Back then, women could not be cared for here in the region. In other words, they basically had to be sent across the republic.’ (gynaecologist working in hospital, 4th interview).*

In summary, when it comes to late abortions, the last step of the process seems to be especially vulnerable for barrier imposing forms of CO, irrespective if they play out on the individual professional – patient – interaction or on a structural level.

## Discussion

The access to abortion services in this specific regional context can currently be considered satisfactory for both early and late abortions based on the results of the study. However, this should not obscure the fact that care is currently provided by a small number of dedicated gynaecologists. Without a regulated use of CO, negative effects are already visible today and future care is uncertain. Throughout our qualitative study it became apparent that early and late abortions both potentially show barrier-imposing manifestations of CO. Structural conditions and resources of abortion services as well as individual interactions between health care professionals and pregnant women seem to be vulnerable to CO. Early and late abortions in this region, however, differ fundamentally in almost all procedural aspects. Differences begin with the general justiciability and the legitimising motives that may or may not be necessary for arranging an abortion, extend to differing local healthcare structures with providers who operate strictly within their own area of service and finally lead to differing practical consequences of CO for women in need.

Barrier-imposing manifestations of CO in the examined region seem to affect mainly the last step of the respective processes in early as well as in late abortions. However, pregnant women might experience these barriers differently: while CO in early abortions plays out especially on a structural level (by further diminishing the number of service providers), women are potentially facing barrier-imposing manifestations of CO also in the individual interaction with a specific doctor in late abortions.

From an ethical point of view, the latter is particularly problematic. While in early abortions the pregnancy conflict is neither initiated nor moderated in the health care system, the situation in late abortions is different. Here, the pregnant woman or the couple are being offered prenatal testing for conditions, where typically no medical treatment is available. Usually, the only option after prenatal testing is to continue with or terminate the pregnancy. Thus, the physician and the health care system do play an important role in the initiation of the pregnancy conflict. It could be argued that physicians, therefore, have more duties in the management of this pregnancy conflict in late abortions. It might seem cynical from the perspective of a pregnant woman, if the same physicians who recommended prenatal testing to her, retreat to CO when it comes to abortion as a consequence. As only few specialized centres offer late abortions, denying the provision of an abortion because of personal motives leaves the women in a difficult situation. Alternative providers are often not immediately available and, again, there is only incomplete information about institutions that perform abortions at all [34].

As mentioned above, access to safe abortion has the primary purpose of promoting autonomy and reproductive self-determination without endangering women’s physical health. Reproductive self-determination as a human right [1] is frequently understood to also encompass access to safe abortion. However, since the right to freedom of thought, conscience and religion is also a human right [2], a dilemma arises. When one right undermines the other and timely and just abortion provision cannot be ensured because of a broad appeal to conscience, measures should be taken to restore a balance [5]. Therefore, a regulatory framework to monitor the practice of CO and restrict its practice if needed should be established. Accordingly, the UN [35] as well as the WHO have criticized the absence of such regulatory frameworks in many countries and further demand states to “ensure compliance with regulations and design/organize health systems to ensure access to and continuity of quality abortion care”. If it proves impossible to adjust the health system so that women’s rights are respected and that timely and safe abortions can be carried out despite CO, “conscientious objection in abortion provision may become indefensible.” [9] (p.28). In addition to improved state regulations with regard to CO, objectors are also called upon to individually fulfil their duty to refer women to doctors who perform abortions in order to enable timely and high-quality abortion provision [9, 36]. Some scholars criticize this approach as an inadequate compromise to balance the conflicting rights as objecting healthcare professionals might include referrals in their objection and view those requirements as complicity in wrongdoing [37]. Mandatory referrals are therefore unlikely to mitigate the effect of CO on abortion provision and are “ineffective in addressing the broader issues with conscientious objection” (p. 360). Thus, the regulatory approach cannot be based merely on an individual appeal for patient referral, but must include the state as a regulatory authority to assess appropriate and effective measures on a domestic level [38]. As de Londras et al. point out in their review, it is the urgent duty of states in which CO is permitted to find a compromise on this issue, despite all the difficulties. In reality, however, legislators often remain silent on this issue or merely formulate unspecific or unclear guidelines. It would be particularly important to provide answers to questions such as who may refuse, what must be done despite refusal and when a refusal on grounds of conscience can be granted at all [39].

That states are willing and able to introduce regulatory measures has already been demonstrated by some European countries. Gynaecologists in Bulgaria, the Czech Republic, Finland and Sweden are not entitled to conscientiously object to the provision of abortion services [17]. In Italy, gynaecologists are obligated to declare their CO formally, which allows the Italian Ministry of Health to collect data of this phenomenon and identify regional gaps in healthcare provision [40]. After a referendum in Portugal in 2007, the hitherto restrictive abortion law was extensively modified [41]. Concerning CO, the law provides that only those professionals directly involved in abortion provision may have the right to object. They have to submit a written declaration that obligates them to refer the woman to a providing colleague and treat the patient if her life is endangered. As the law also requires hospitals to guarantee abortion access, CO is not seen as a barrier to abortion services in Portugal [15, 41]. Comparable guidelines or measures are still missing in Germany. However, it is not a matter of solely establishing laws and guidelines; these must also be implemented and monitored. Based on our experts’ insights, one effective way could be to not only require a formal declaration of CO but also obligatorily involve hospitals in abortion services. Not only would such a regulation counter potential barriers on a structural level, but also prevent CO from having a negative impact on the women on the individual level during the medical encounter as processes of referral are much easier in a larger institution and team. In addition, more students and young doctors would become familiar with abortion services during training and, thus, have a chance to reflect more profoundly upon their own professional and personal preferences and values in relation to these services.

Are there alternative approaches to dealing with barrier-imposing manifestations of CO? In the Netherlands, abortions are legal on request if performed before the 24th week of pregnancy. Abortions are only performed in licensed and specialised abortion clinics irrespective if they are early or later in pregnancy [42]. Very late-term abortions after the 24th week of pregnancy are only possible if there are serious medical reasons, e.g. if the newborn has a condition, or a combination of conditions, that is incompatible with life [43]. Such abortions also take place in hospitals. It has been reported that abortions in the Netherlands are” safe” and “easily available” [44]. As highlighted by the European Abortion Access Project, CO “does not seem to affect access to abortion care, because most abortions are performed in private clinic subsidized by the State specializing in abortion care, and in these clinics there are no objectors.” [45]. Creating central contact points for the performance of abortions hence appears to be an effective way to ensure the availability of and access to abortions without restricting the right to CO outside these providing institutions.

## Strengths and limitations

Our study design was based on a series of qualitative interviews with stakeholders of all instances involved. We aimed to give a voice to every professional group that bears responsibility in the process of early as well as late-term abortions in the examined region. Through this multi-perspectivity, we hoped to develop an accurate picture of how abortions are handled in one specific regional context and at which levels problems can arise due to CO from the health care providers’ perspective. Thereby, we have carried out the first study of its kind in Germany which we are aware of. Nevertheless, a larger number of interviews would have been necessary to obtain comprehensive results. Accordingly, the findings derived from the interviews should be interpreted in the context of such a limitation. Further limitations are of methodological nature and arise from the study approach. Given the social and political controversy of the topic, interviewees may have been reluctant to divulge any sensible information face-to-face. Furthermore, as no conscientious objectors were included in the recruitment of interview participants, their views and perceptions of the processes were underrepresented. Likewise, the experiences and perspectives of affected women and families that were confronted with CO while seeking abortion have not been included in this study, as only health care workers were interviewed. Since the overall goal in this context is to improve healthcare for pregnant women, it is particularly important to include their experiences in the process to develop a just, equal and non-discriminatory health system [46]. Finally, our findings are limited to a specific regional context and might not be indicative for other parts of Germany.

## Conclusion

Conclusively, our findings indicate that CO possibly imposes barriers to both early and late abortion provision and especially in the last procedural steps. The barriers could manifest in individual professional-patient interactions as well as on a structural level. Further regional and national studies are needed to verify our findings. To oblige hospitals to partake in abortion provision in Germany has the potential to prevent negative impacts of CO on women’s rights on an individual as well as on a structural level.

### Supplementary Information

**Additional file 1.** Interview guide for semi-structrued interviews.

## Data Availability

The datasets generated and analysed during the current study are not publicly available due to the anonymity promised to the interview participants but are available from the corresponding author on reasonable request.
