# Supplementary material for: Conscientious objection and barriers to abortion within a specific regional context - an expert interview study
Source: BMC Med Ethics. 2024 Feb 6;25:14. doi: 10.1186/s12910-024-01007-1 (PMC10848386; doi:10.1186/s12910-024-01007-1)
Supplement: Supplementary file 1 — Additional file 1. Interview guide for semi-structrued interviews. [file 12910_2024_1007_MOESM1_ESM.zip › Box_Legal Framework in Germany.docx]

**Legal Framework in Germany**

The German legal situation regarding abortions dates back to the 19th century [1]. Over the years, the paragraphs on abortion in the German Penal Code (§218 and §219 StGB) were repeatedly adjusted, but, in principle, remained the same. As §218 StGB states, abortions are illegal. However, women seeking abortion at own request remain exempt from prosecution if certain prerequisites are met. Inter alia, the procedure must be performed by a physician before the 12^th^ week of pregnancy post conceptionem (p.c.) and a 3-day reflection period must be observed after the woman has attended mandatory psychosocial counselling by a certified counselling centre (early abortion). In contrast, an abortion is legal if it is necessary to avert a danger to the life or the physical or mental health of the pregnant woman (“medical indication”). Such a medical indication is usually given after prenatal testing has confirmed an incurable disease or chromosomal aberration in the foetus. In such cases, abortion is theoretically possible until the end of the pregnancy (late abortion).

In addition to the excerpts from the German Criminal Code, the Act on Assistance to Avoid and Cope with Conflicts in Pregnancy (German abbreviation: SchKG) regulates the provision of abortions. Section 12 is the only official regulation on the use of CO in the context of abortion in German law: “no person shall be obliged to take part in an abortion. [This] shall not apply if participa­tion is necessary to avert an otherwise un­avoidable risk to the life of the pregnant woman or of grave injury to her health.”

References:

1. David HP, Fleischhacker J, Hohn C. Abortion and eugenics in Nazi Germany. Popul Dev Rev. 1988;14(1):81-112. doi:<https://doi.org/1972501>
