# Supplementary material for: Conscientious objection and barriers to abortion within a specific regional context - an expert interview study
Source: BMC Med Ethics. 2024 Feb 6;25:14. doi: 10.1186/s12910-024-01007-1 (PMC10848386; doi:10.1186/s12910-024-01007-1)
Supplement: Supplementary file 1 — Additional file 1. Interview guide for semi-structrued interviews. [file 12910_2024_1007_MOESM1_ESM.zip › Interview guide for semi-structrued interviews (english).docx]

| Main questions | Additional questions | Clarifying questions |
| --- | --- | --- |
| 1) Can you please introduce yourself and your field of work? | - How do you come into contact with abortions in your professional practice? | - Can you please clarify what you meant by …? - Can you please expand a little on …? - Can you please give an example for …? - In particular, what do you think of …? |
| 2) Could you outline what the typical pathway of an early abortion according to the counselling regulation looks like in the city? | - Who do women initially contact for help? - How do women receive information about where the first contact should take place? - Who performs early abortions in the city? - How many registered doctors and clinicians perform abortions in the city? - Is it necessary for registered doctors and hospitals to cooperate to perform abortions? - Which abortion methods are mainly used? - Where do women receive post procedural aftercare? - To what extent are these processes also influenced and guided by local politics? |  |
| 3) How do the pathways which you have just explained differ for late abortions after the 12^th^ week of pregnancy? | - Who performs late abortions in the city? - How many of the doctors perform late abortions? How does this number differ from those performing early abortions? - How do you assess the medical care situation with regard to both early and late abortions in the city? |  |
| 4) Are there alternative pathways for women who decide to have an abortion? | - In which proportion do pregnant women choose to go to neighbouring countries with more liberal legal systems? - Which factors may affect the decision for women to go abroad? |  |
| 5) In which ways may CO to abortion influence the medical care situation of women in the city? | - How do you assess the counselling of pregnant women regarding their options, even from objecting medical professionals? - Do objecting medical professionals always act according to existing guidelines (e.g. timely referral)? - Which other factors in the realm of conscientious objection (e.g. institutional objection) affect abortion performance? - Do conscientious objections affect timely treatment of women? |  |
| 6) In your opinion, are political actions needed in Germany to ensure adequate medical care for women against the background of CO? | - Should legal changes be sought or rather further work on existing guidelines? |  |

**Appendix: Interview guide for semi-structured interviews**

**Additional questions:**

- What do you personally know about conscientious objection?
  - How do you define it?
  - What do you think, which professional groups have the right to conscientiously object?
  - Who controls the guideline-compliant implementation of CO?
- Which role do moral concerns of medical professionals and their personal attitudes towards abortion play in medical practice?
- Do medical professionals who perform abortions have to fear personal, professional or other consequences?
  - How does the willingness to perform abortions affect the workload of medical professionals?
  - To what extent does the willingness to perform abortions influence career opportunities?
  - How do these unpleasant side effects affect their willingness to perform abortions?
